# Society Meetings

**Published:** 1911-01

**Authors:** 


### SOCIETY MEETINGS

---

The Medical Society of the State of New York will hold its one hundred and fifth annual meeting at Albany, Monday, Tuesday, and Wednesday, January 30, 31 and February 1, 1911, under the presidency of Dr. Charles Stover, of Amsterdam. The house of delegates will meet Monday evening, and the regular scientific work will begin Tuesday morning. Up to the time of printing the Journal, the program had not been received.

---

The American Academy of Medicine held its midwinter meeting in Buffalo, December 1, and 2, 1910. Sociologics and economics relating to hospitals were the principal topics of discussion. Charles W. Pardee, president of the Board of Managers of the Buffalo General Hospital read a paper the second day entitled: "On the Relation of the Hospital to the Community."

Other papers were read on similar lines by Drs. Edward B. Heckel and Ira S. Wile of New York, Dr. Edgar A. Vander Veer of Albany, Dr. Frank Dow of Rochester, Dr. John L. Heffron of Syracuse, Dr. Arthur W. Hurd of the Buffalo State Hospital, Frederic Almy of the Charity Organization Society and John R. Shilliday of the Buffalo Society for the Relief and Control of Tuberculosis.

---

The National Confederation of State Medical Examining and Licensing Boards will hold its twenty-first annual meeting in Chicago, Ill., on Tuesday, February 28, 1911, at the Congress Hotel.

The subjects to be taken up at this meeting will be a consideration of the state control of medical colleges; a report by a special committee on clinical instruction; a report on a proposed materia medica list by a special committee; the report on a paper presented at the St. Louis meeting by Mr. Abraham Flexner of the Carnegie Foundation for the Advancement of Teaching; and some special papers on such subjects as the regulation of medical colleges, necessity for establishing a rational curriculum for the medical degree, and others, by men eminently qualified to deal with these subjects.

The symposium will be composed of ten papers to be presented from the viewpoints of state, law, medical colleges, state medical examining and licensing boards. The chief object of the symposium is to determine, as far as possible, the feasibility of placing medical colleges under state control. The special committee on materia medica made a report at the St. Louis meeting of the Confederation June 6, 1910, and it was continued and instructed to report again at the next annual meeting of the Confederation in 1911. The report of this committee made at St. Louis has received favorable comment by many of the editors of medical journals and should receive at the Chicago meeting extended and careful consideration. The report on Mr. Flexner's paper is published in the proceedings of the St. Louis meeting of the Confederation, page 64, and will be open for discussion at the Chicago meeting.

An earnest and cordial invitation to this meeting is extended to all members of State medical examining and licensing boards, teachers in medical schools, colleges and universities, delegates to the Association of American Medical Colleges, to the council on medical education of the A. M. A., and to all others interested in securing the best results in medical education.

The officers of the Confederation are, president, J. C. Guernsey, 1923 Chestnut Street, Philadelphia; secretary-treasurer, George H. Matson, State House, Columbus, Ohio.

---

THE Rochester Academy of Medicine held meetings during December as follows:

*Section of General Medicine.*—Wednesday evening, December 7. Program: Suboxidation as a Factor in the Causation of Disease, Mary E. Dickinson.

*Section of Surgery.*—Wednesday evening, December 14. Program: Decompression, Charles T. Frasier, Philadelphia. Discussion opened by Edgar R. McGuire, Buffalo.

---

THE Buffalo Academy of Medicine held meetings during November and December as follows:

*Section of Obstetrics and Gynecology.*—Tuesday evening, November 29. Program: Views on the treatment of puerperal septic infection; past and present, James E. King.

*Section of Surgery.*—Tuesday evening, December 6. Program: Technic of treatment of appendicitis, Alexander B. Johnson, Attending Surgeon of the New York Hospital.

*Section of Medicine.*—Tuesday evening, December 13. Program: Theodore Schwann, Biologist, Physiologist. An appreciation in commemoration of the centennary of his birth, Frederick C. Busch; Diagnosis of gastro-intestinal lesions by radiography, Lewis Gregory Cole. New York. Discussion: From the surgeon's viewpoint, Roswell Park and Earl P. Lothrop; from the radiographer's viewpoint, W. Ward Plummer and Leonard Reu; from the internist's viewpoint, Henry C. Buswell and DeLancey Rochester.

---

THE Medical Society of the County of Chemung, held its annual meeting in the society rooms in the Federation Building, Elmira, Tuesday, December 20, 1910, at 3.00 P. M. The program presented the following papers: Some unexpected experiences in lobar pneumonia, Charles G. Stockton, Buffalo; Notes on a case of manic depressive insanity, C. J. Patterson, Owego.

Election of officers for 1911 was held.

The president was E. T. Bush, U. B., 1904; and Charles Haase, U. B., 1902, was secretary.

---

THE Medical Society of the County of Erie held its annual meeting in the rooms of the Society of Natural Sciences, Buffalo Library Building, Monday, December 19, 1910, at 8 p. m.

The following order of business was arranged:

(1) reading minutes of previous meeting and consideration of amendments to by-laws; (2), report of council; (3), election of officers; (4), address of retiring president, Grover W. Wende; (5) report of secretary, F. C. Gram; (6) report of treasurer, A. T. Lytle; (7), report of committee on legislation, F. Park Lewis; (8), report of committee on public health, Henry Reed Hopkins; (9), report of committee on membership, T. H. McKee; (10), report of committee on necrology, H. J. Mulford; (11), report of milk commission, Charles Sumner Jones; (12) report of commission on contagious diseases hospital, J. D. Bonnar; (13), report of committee on division of fees, J. H. Pryor.

The election of officers resulted as follows: president, Daniel V. McClure; first vice-president, Thomas H. McKee; secretary, Franklin C. Gram; treasurer, A. T. Lytle; board of censors, John H. Grant, Francis E. Fronczak, A. G. Bennett, G. L. Brown,

Lawrence H. Hendee; delegates to the Medical Society of the State of New York, John H. Pryor, F. C. Busch, Edward Clark, George J. Eckel, Eli H. Long; chairmen of committees, legislation, F. Park Lewis; public health, Henry R. Hopkins; membership, Charles A. Wall.

